# Genetic and epidemic characteristics of porcine parvovirus 7 in the Fujian and Guangdong regions of southern China

**DOI:** 10.3389/fvets.2022.949764

**Published:** 2022-08-17

**Authors:** Xinjie Zhang, Canyang Zheng, Zixin Lv, Shaohua Xue, Yuxuan Chen, Yanru Liu, Xirong Huang, Guoqing Luo, Xiaoyan Yang, Ailing Dai

**Affiliations:** ^1^College of Life Sciences, Longyan University, Longyan, China; ^2^Fujian Engineering Research Center for Swine Disease Control and Prevention, Longyan, China; ^3^Fujian Provincial Key Laboratory for the Prevention and Control of Animal Infectious Diseases and Biotechnology, Longyan, China

**Keywords:** porcine parvovirus 7, genotype, molecular characterization, coinfections, recombination

## Abstract

Porcine parvovirus (PPV) is the primary cause of reproductive disorders in pigs. The porcine parvovirus 7 (PPV7) subtype was first identified in the United States in 2016. In this study, PPV7 was detected in different porcine samples, including serum, feces, saliva, and milk, from 69 pig farms in the Fujian and Guangdong regions of South China, and its coinfection with porcine circovirus 2 (PCV2), porcine circovirus 3 (PCV3), and porcine reproductive and respiratory syndrome virus (PRRSV) was determined. Whole-genome sequencing, phylogenetic analysis, and recombination analysis were performed on seven isolates, with each selected isolate originating from a different farm. There was a high rate of PPV7 positivity in blood, stool, and saliva but PPV7 DNA was absent from breast milk. The findings also showed that PPV7-positive samples had a high rate of coinfection with PCV2, PCV3, and PRRSV. Real-time PCR was used to determine the viral copy numbers of PCV2, PCV3, PRRSV, and PPV7 in serum samples and to assess whether PPV7 affected PCV2, PCV3, and PRRSV viral loads. Phylogenetic analysis showed that PPV7e and PPV7f were the most prevalent and widespread subtypes in the Fujian and Guangdong regions, respectively. While the PPV7a, PPV7b, PPV7c, and PPV7f subtypes were most prevalent in Fujian Province, PPV7a-e subtypes were prevalent in Guangdong, indicating that PPV7 has rich genetic diversity in these regions. A putative recombinant strain, 21FJ09, was identified using SimPlot and the Recombination Detection Program 4 software.

## Introduction

*Parvoviridae* are small, non-enveloped, single-strand linear DNA viruses with 4–6 kb genomes that contain two major open reading frames (1). According to the virus classification and taxonomic proposal approved by the International Committee on Taxonomy of Viruses (2), the *Parvoviridae* family is classified into three subfamilies: *Parvovirinae, Hamaparvovirinae*, and *Densovirinae*. Viruses infecting vertebrate and invertebrate hosts are assigned to *Parvovirinae* and *Densovirinae*, respectively, while *Hamaparvovirinae* has a broad host range that includes both vertebrates and invertebrates (http://talk.ictvonline.org/taxonomy/). To date, seven porcine parvovirus (PPV) genotypes of *Parvovirinae* and *Hamaparvovirinae* are reported in pigs. These belong to four genera, *Protoparvovirus* (PPV1), *Tetraparvovirus* (PPV2–3), and *Copiparvovirus* (PPV4–6) of the *Parvovirinae* subfamily and *Chaphamaparvovirus* (PPV7) of the *Hamaparvovirinae* subfamily (1–3).

PPV is a major swine pathogen that can cause recurring estrus, abortion, and the delivery of mummified or stillborn fetuses, commonly described by the acronym SMEDI (stillbirth, mummification, embryonic death, and infertility) (4). PPV1 was first isolated in 1965 from a cell culture contaminant in Germany (5). PPV2 was originally detected in Myanmar in 2001 and identified as a possible factor for porcine respiratory disease complex (6, 7). In 2008, PPV3 was first discovered in Hong Kong, China (8). Both PPV4 and PPV5 were identified in 2010 and 2013, respectively (9, 10), in the United States, and PPV6 was first identified in 2014 in China using metagenomic sequencing of aborted pig fetuses (3). PPV7 was first identified in 2016 in the United States using metagenomic sequencing of rectal swabs from healthy adult pigs (11). PPV7 was later found in China, first in Guangdong Province (12), and then in Anhui, Guangxi, Fujian, Jiangsu, Shandong, and Hebei provinces (13–15). This virus was subsequently shown to infect the fetus through semen and be a potential cause of reproductive failure among sows (16). Studies have also reported the coinfection of PPV7 with other swine pathogens including PCV2 and PCV3 (13, 17). The coinfection of PPV7 and PCV3 was found to be significantly higher in serum from sows that experienced reproductive failure (17). Thus, it is proposed that PPV7 may cause disease in pigs in combination with PCV2 or PCV3. However, the pathogenicity and distribution of PPV7 among pig herds in regions of southern China remain unknown.

This study investigated the prevalence of PPV7 in Fujian and Guangdong and examined whether this virus could be transmitted through breast milk. Coinfection of PPV7 with PCV2, PCV3, and porcine reproductive and respiratory syndrome virus (PRRSV) was also assessed, and full-genome sequencing of PPV7 strains isolated from the Fujian and Guangdong provinces was performed.

## Materials and methods

### Sample collection

From March 2021 to April 2022, 500 serum samples from sows with fever were collected from 72 pig farms in Guangdong (*n* = 146) and Fujian (*n* = 354). Fecal samples (*n* = 373) were also collected from PPV7-positive farms, 114 and 259 of which were from pigs with clinical symptoms of diarrhea and healthy pigs, respectively. Saliva samples (*n* = 218) were collected from PPV7-positive farms and 76 milk samples from healthy sows were obtained from PPV7-positive farms in Fujian. Detailed information about the samples is listed in Table 1. After collection, the samples were immediately transported to the laboratory at 4°C and stored at −80°C.

**Table 1:** Sample details.

**Region**		**Serum**	**Fecal of diarrhea**	**Fecal of healthy**	**Saliva**	**Milk**
Guangdong	Qingyuan	5/30 (2/3)	0 (0)	0 (0)	0 (0)	0 (0)
	Huizhou	3/24 (1/2)	0 (0)	9/35 (1/1)	0 (0)	0 (0)
	Meizhou	7/24 (4/4)	2/24 (1/1)	0 (0)	0 (0)	0 (0)
	Shantou	25/68 (3/3)	0 (0)	0 (0)	0 (0)	0 (0)
Fujian	Longyan	36/137 (16/24)	4/30 (5/5)	31/104 (7/7)	15/107 (1/1)	0/40 (1/1)
	Sanming	16/63 (5/11)	0/21 (1/1)	16/63 (3/3)	0 (0)	0 (0)
	Zhangzhou	23/106 (6/17)	3/32 (3/3)	2/17 (1/1)	23/111 (1/1)	0/36 (1/1)
	Nanping	6/48 (3/8)	0/7 (1/1)	7/40 (2/2)	0 (0)	0 (0)

The proportion of PPV7 positive samples in each region; the values in parentheses indicate the proportion of positive pig farms. All feces, saliva, and milk samples were collected from PPV7 positive farms.

### Pathogen DNA extraction and real-time PCR detection

Viral DNA and RNA were extracted from serum, fecal, saliva, and milk samples using the Virus Genome Extraction Kit (Magen Biotech, Guangzhou, China) according to the manufacturer's instructions. Duplex real-time PCR assays were performed to detect PCV2 and PCV3 (18). PRRSV and PPV7 were detected using a single real-time PCR assay (19, 20). Samples with Ct values ≥37 were considered negative. Copy numbers of viral genomic DNA extracted from samples were determined using a standard curve.

### Statistical analyses

All statistical analyses were performed using GraphPad Prism version 5.0.0 for Windows (GraphPad Software, San Diego, CA, USA) and SPSS 27.0 software (SPSS Inc., Chicago, IL, USA). Differences in PCV2, PCV3, and PRRSV prevalence rates among different PPV7 serum categories were assessed using Fisher's exact test by pairwise comparisons. One-way ANOVA was used to evaluate the relationships between PCV2, PCV3, PRRSV status, and PPV7 viremia level expressed as copy numbers. The linear correlation between the viral load of PPV7, PCV2, PCV3, and PRRSV-positive samples was also assessed. Statistical significance was set at *p* < 0.05, and confidence intervals were calculated.

### Amplification and sequencing of complete PPV7 isolates

PPV7-positive samples from different farms were used for genome amplification with three primer pairs (P1–P3) reported for genome amplification (15). The PPV7 genome was amplified using 2x Phanta Master Mix (Vazyme, Nanjing, China) according to the manufacturer's instructions. PCR was performed using the following protocol: (1) initial denaturation at 95°C for 3 min and (2) 35 cycles at 95°C for 15 s, 55°C for 15 s, and 72°C for 1.5 min. The PCR products were separated using 2% agarose gel electrophoresis and cloned into a pESI-blunt vector (Yeasen, Shanghai, China). The positive clones were screened using PCR and sent to a commercial facility (Rui Biotech, Guangzhou, China) for sequencing. Sequences were assembled and edited using Seqman (DNASTAR, Madison, WI, USA).

### Sequence alignment and phylogenetic analysis

The nucleotide sequence homology between the PPV7 isolates and other reference strains was assessed using the ClustalW package in MegAlign (DNASTAR, Madison, WI, USA). The best fit nucleotide substitution model was selected using ModeFind in IQ-tree (21). Phylogenetic trees were constructed using MEGA 7.0, and the maximum-likelihood (ML) method was used to strengthen the robustness of the results. The pre-estimated General Time Reversible distribution model (GTR), with 1,000 bootstrap replicates, was used for nucleotide substitution.

### Recombination analysis

Recombination evaluations were conducted for the isolates and 121 PPV7 genomes available in the NCBI database. All genome sequences were screened with Recombination Detection Program 4 (RDP4, version 4.101) using the RDP, GENECONV, Chimera, MaxChi, BootScan, SiScan, and 3Seq packages. Recombinant events identified using the RDP4 software (version 4.101) were further analyzed using SimPlot software (version 3.5.1). Recombination events in multiple sequence alignments were identified using BootScan analysis in SimPlot (version 3.5.1). The window size was 200 bp, and a step size of 20 bp was chosen between plots.

## Results

### PPV7 detection

PPV7 DNA was detected in serum, feces, and saliva collected from pigs in Fujian and Guangdong Provinces. The presence of virus in serum, feces, and saliva was 24.20% (121/500), 19.84% (74/373), and 17.43% (38/218), respectively (Table 1). Interestingly, the PPV7 positivity rate was 25.10% (65/259) in the feces of healthy porcine and 7.89% (9/114) in the feces of pigs with diarrhea. PPV7 DNA was absent from the milk samples of healthy sows.

PPV7 frequently coinfects pigs along with PCV2, PCV3, and PRRSV, and induces viremia. In the current study, PCV2 was detected in 51.00% (255/500), PCV3 in 32.40% (162/500), and PRRSV in 11.40% (57/500) of the serum samples from sows with fever symptoms. PPV7 had a coinfection rate of 14.00% (70/500) with PCV2, 9.00% (45/500) with PCV3, and 4.60% (23/500) with PRRSV. In the 121 serum samples categorized as POS-PPV7, the PCV2, PCV3, and PRRSV positivity rates were 57.85% (70/121), 37.19% (45/121), and 19.01% (23/121), respectively. In the 379 serum samples categorized as NEG-PPV7, the PCV2, PCV3, and PRRSV positivity rates were only 48.81% (185/379), 30.87% (117/379), and 8.97% (34/379), respectively.

### PCV2, PCV3, and PRRSV viremia is higher in PPV7-positive pigs

**Figure 1:**
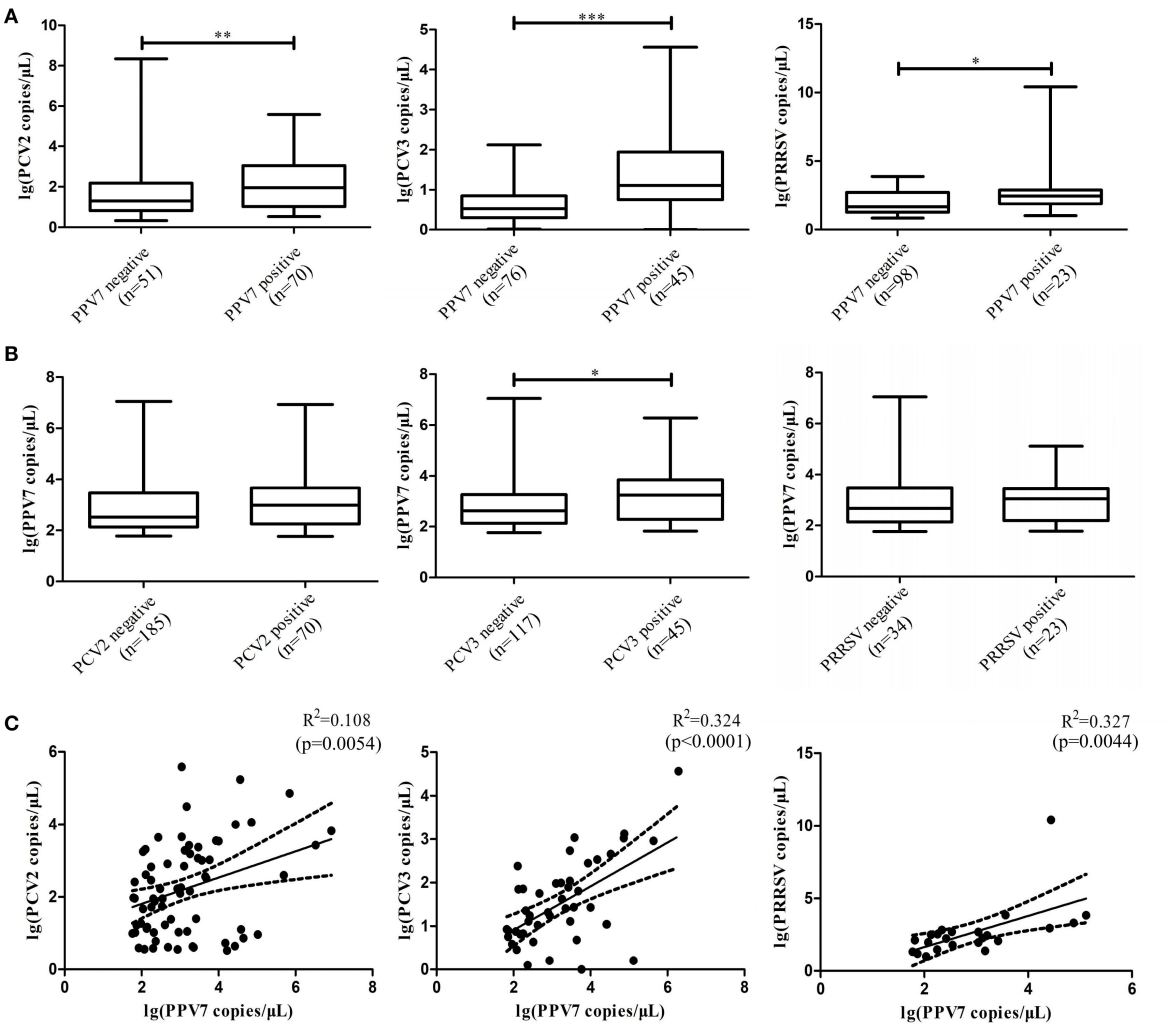
Boxplot comparison of the real-time PCR copy number of PPV7 with PCV2, PCV3, and PRRSV, and scatterplots with the trends and 95% CI (dashed line) for the copy number of PPV7 with PCV2, PCV3, and PRRSV. **(A)** Comparison of the real-time PCR copy number of PCV2, PCV3, and PRRSV in POS-PPV7 and NEG-PPV7 serum samples. **(B)** Comparison of the real-time PCR copy number of PPV7 in POS-PCV2, NEG-PCV2, POS-PCV3, NEG-PCV3, POS-PRRSV, and NEG-PRRSV serum samples. Statistically significant (*p* < 0.05) differences are marked with an asterisk. **(C)** Scatterplots with the trends and 95% CI (dashed line) for the copy number of PPV7 with PCV2 (*p* = *0.0054*), PCV3 (*p* < 0.0001) and PRRSV (*p* = 0.0044).

To evaluate the impact of PPV7 coinfection with PCV2, PCV3, and PRRSV on viremia, PPV7, PCV2, PCV3, and PRRSV copy numbers in serum samples were detected using real-time PCR. Copy numbers were significantly higher in the POS-PPV7 than in the NEG-PPV7 serum samples (*p* = 0.0097, *p* < 0.0001, *p* = 0.0363, respectively) (Figure 1A). PCV2 and PRRSV are shown to cause immunosuppression during infection. Interestingly, the reverse comparison showed that PPV7 copy numbers were not significantly different between the POS-PCV2/NEG-PCV2 and POS-PRRSV/NEG-PRRSV serum samples. Notably, the PPV7 copy number was higher in POS-PCV3 samples (*p* = 0.0398) than in NEG-PCV3 samples (Figure 1B). Meanwhile, the correlation between the PPV7 copy number in serum samples positive for PPV7 and PCV2, PCV3, PRRSV was statistically significant (*p* = 0.0054, *p* < 0.0001, *p* = 0.0044, respectively). The linear correlation coefficient (*r*) between PPV7 and the PCV2, PCV3, and PRRSV copy numbers was 0.329, 0.569, and 0.572, respectively (Figure 1C). The square of correlation (*r*^2^) scores were 0.108, 0.324, and 0.327, respectively, thus the PPV7 copy number could account for 10.8, 32.4, and 32.7% of the PCV2, PCV3, and PRRSV copy numbers.

### Sequencing and phylogenetic analysis

**Figure 2:**
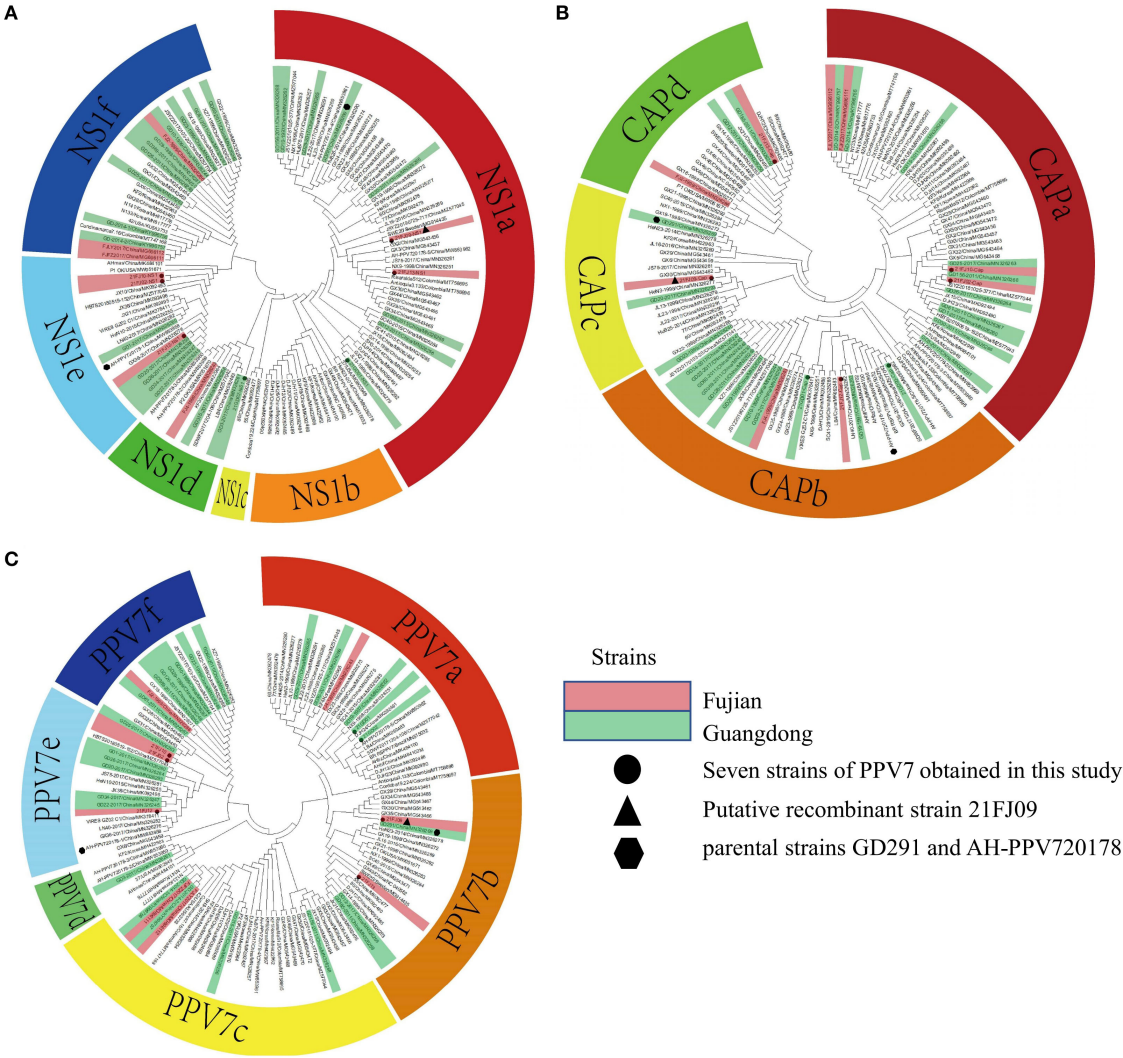
Phylogenetic analysis of the nucleotide sequences of PPV7. The trees were constructed based on the *NS1* **(A)**, *cap* **(B)**, and complete genome sequences **(C)** by the maximum-likelihood (ML) phylogenetic method, using the General Time Reversible model. The analysis involved 128 nucleotide sequences, including seven sequences obtained from this study and 121 reference strains obtained from the NCBI database. The circles represent the seven strains from this study, the triangles represent the putative recombinant 21FJ09 strain and the hexagons represent the parental strains, GD291 and AH-PPV720178-1. The strains from Fujian and Guangdong are marked as red and green, respectively.

Seven complete PPV7 genome sequences from Guangdong (*n* = 2) and Fujian (*n* = 5) provinces were amplified using PCR (Table 2). All complete genomes of PPV7 obtained in this study were 3984–3999 nt in length, and 7 *NS1* sequences were 2019 nt in length. There were no deletions or insertions, however, three *cap* gene lengths of 1,425 nt, 1,422 nt, and 1,410 nt were found among the seven *cap* sequences that were obtained. Based on the nucleotide similarity of the coding region, the seven sequences shared 92.9–97.5% similarity, including 92.9–97.5% similarity in *NS1 gene* and 87.4–98.5% similarity in *cap* gene. These seven sequences shared 90.1–97.1% genomic similarity with the reference strain, including 92.6–97.8% similarity with the *NS1* gene, and 85.5–99.0% similarity with the *cap* gene. To better understand the variation and genetic characteristics of PPV7 in the Guangdong and Fujian regions during the study period, 121 complete or nearly complete PPV7 genome sequences from six countries were obtained from GenBank. These sequences, along with the PPV7 sequences obtained from this study, were used to reconstruct phylogenetic trees using the *NS1* and *cap* genes as well as the complete genome. According to the *NS1*-based phylogenetic analyses, the 128 *NS1* sequences from PPV7 can be classified into six main clades: NS1a–f (Figure 2A). While the NS1a, NS1d, NS1e, and NS1f subtypes were found in Fujian, all six NS1a–f subtypes were found in Guangdong. According to the *cap*-based phylogenetic analysis, the 128 *cap* gene sequences were classified into four CAPa-d subtypes, all of which were prevalent in Fujian and Guangdong (Figure 2B). Based on the maximum likelihood phylogenetic analysis of 128 complete genomes, PPV7 fell into six subtypes, PPV7a-f (Figure 2C). The complete gene sequences of nine strains in the Fujian Province (including the five strains obtained in this study) were classified into five subtypes, PPV7a, PPV7b, PPV7c, PPV7e, and PPV7f. PPV7e was the most prevalent, accounting for 33.33%, followed by the PPV7b, PPV7c, PPV7a, and PPV7f subtypes, accounting for 22.22, 22.22, 11.11, and 11.11%, respectively. The complete gene sequences of 26 strains from Guangdong Province (including the two strains obtained in this study) were classified into all six subtypes, PPV7a-f, approximately 30.77% of which belonged to PPV7f. PPV7a-e accounted for 19.23, 11.54, 15.38, 3.85 and 19.23% of the strains in this region, respectively.

**Table 2:** The PPV7 strains identified in this study.

**No**.	**Strains**	**Genotype**	**Sample type**	**Source**	**GenBank accession no**.
1	21FJ02	PPV7f	Serum	Longyan	ON462331
2	21FJ09	PPV7a	Serum	Longyan	ON462332
3	21FJ10	PPV7f	Serum	Zhangzhou	ON462333
4	21FJ12	PPV7a	Serum	Nanping	ON462334
5	21FJ13	PPV7c	Serum	Sanming	ON462335
6	21GD01	PPV7d	Serum	Meizhou	ON462336
7	21GD02	PPV7d	Serum	Shantou	ON462337

### Recombination analysis

**Figure 3:**
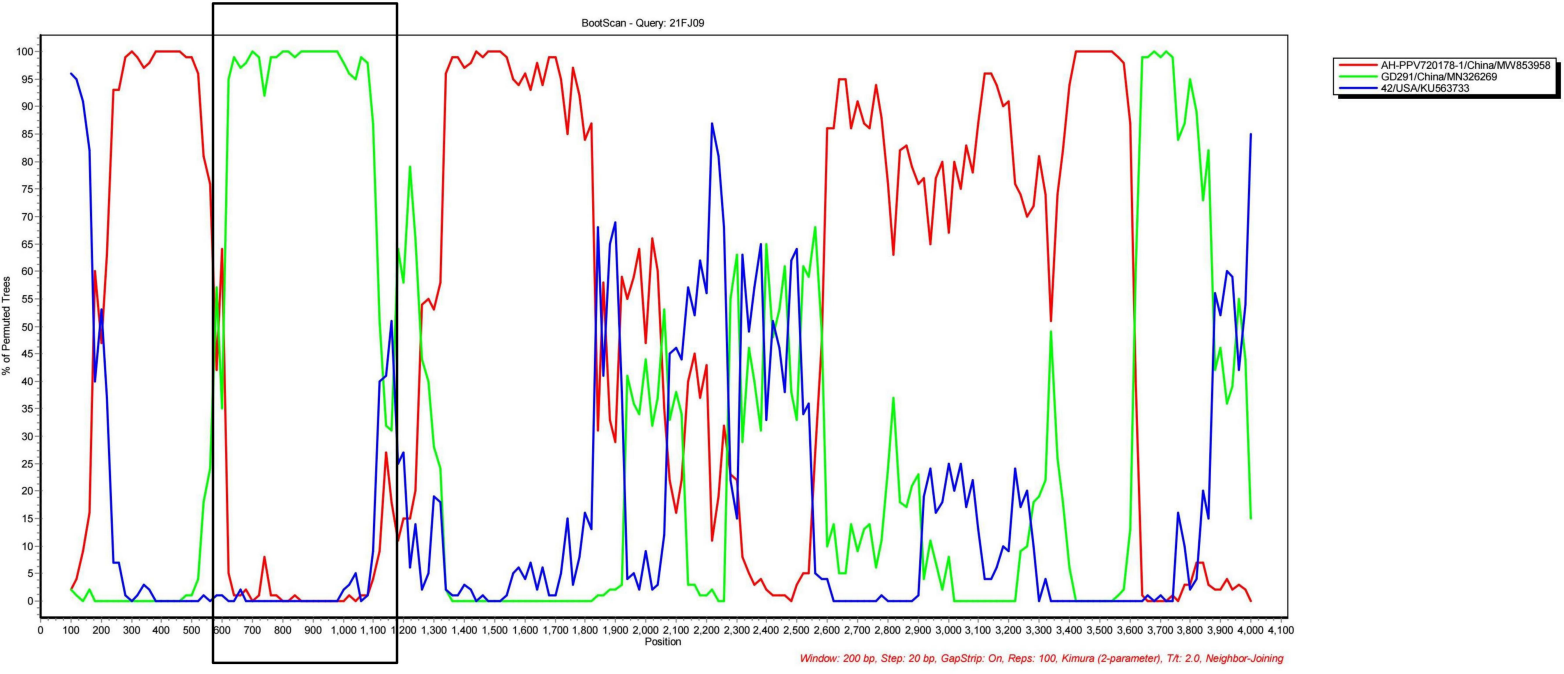
SIMPLOT recombination analysis. Bootscanning analysis was performed using 21FJ09, GD291, AH-PPV720178-1, and 21FJ09 as query sequences, with a sliding window of 200 nt that moved in 20 nt steps.

**Figure 4:**
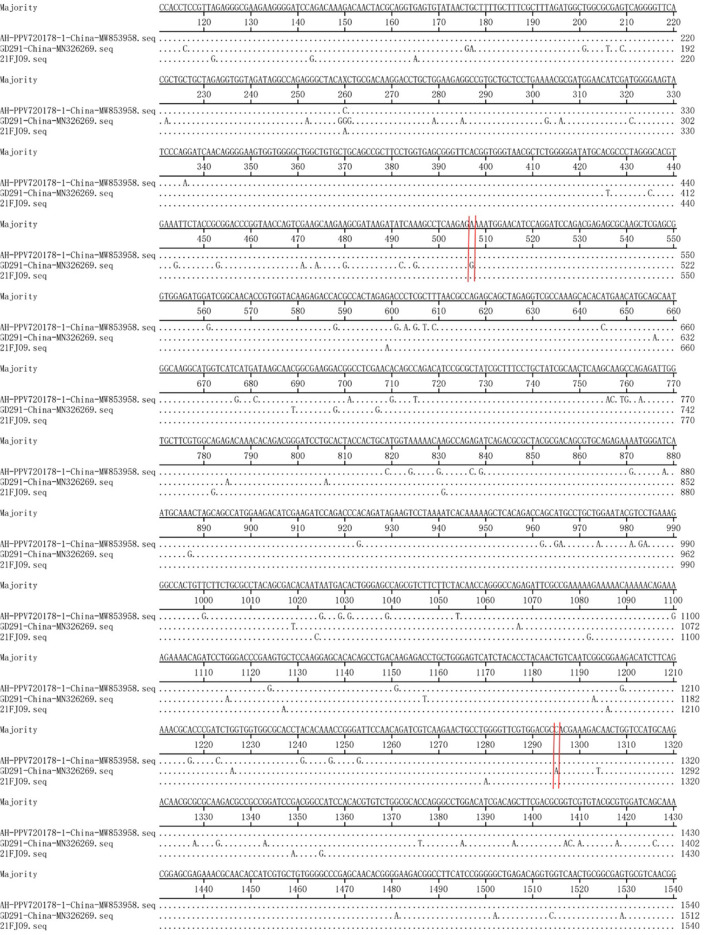
Base-by-base comparison of the recombination fragment of the 21FJ09 isolate and probable parents (GD291 and AH-PPV720178-1). The position of the potential breakpoint is bracketed by two vertical lines.

Recombination evaluations were conducted for the seven isolates and 121 PPV7 genomes obtained from the GenBank database. All the genome sequences were screened with RDP4 using the RDP, GENECONV, Chimera, MaxChi, BootScan, SiScan, and 3Seq packages. The results showed that the 21FJ09 strain was a putative recombinant. The genetic relationship between the putative recombinants and major or minor parents in the different genome segments is shown in Figure 2, indicating that the recombination events could occur between clades. In this inferred recombination event, GD291 and AH-PPV720178-1 were the major and minor parental strains of the 21FJ09 strain, respectively. SimPlot 3.5.1 software was used to further evaluate the two recombinants and their major and minor parental strains (Figure 3). The inferred recombinant event of 21FJ09 (region 507–1295 nt) primarily occurred from the 5′UTR to the N-terminal of the *NS1* gene (Figure 4).

## Discussion

PPV causes major reproductive failure in sows and is an economically important viral pathogen for the global pig industry. PPV subtypes usually coinfect along with PCV2 and may play an associated role in disease development (22). PPV7 is a novel PPV that was recently identified and shown to be infectious, suggesting that it may pose a threat to the pig industry. To date, the virus has been detected in serum, fecal swabs, nasal swabs, and lung lavage, suggesting that transmission of this virus may occur in a variety of ways (11). In this study, 500 serum samples from diseased pigs and 373 fecal swabs (259 from healthy pigs and 114 from pigs with diarrhea) from Fujian and Guangdong swineherds were examined. The high positivity rate of PPV7 in the serum and fecal swabs suggested that this subtype is widespread in Guangdong, Fujian, and even throughout east and south China. Interestingly, the positivity rate of PPV7 was significantly higher in the fecal swabs of healthy pigs than in the fecal swabs of pigs with diarrhea. However, despite its prevalence in fecal swabs, this virus is not associated with diarrhea in pigs. Given that PPV7 also infects saliva samples, this may be an important mode of transmission. In addition, there is evidence that severe acute respiratory syndrome coronavirus 2 (SARS-CoV2) RNA is present in human milk, but whether milk promotes transmission requires further research (23). While the specific deleterious effects of PPV7 infection in piglets remain unknown, PPV1 causes very clear harm to piglets and the pig industry. Given that PPV7 has the potential to become the next PPV1, it is important to investigate whether PPV7 can be transmitted through breast milk in order to inform the development of prevention and control measures. However, this study found no evidence of PPV7 in milk. Thus, these findings do not support mother-to-infant transmission of PPV7 *via* milk.

PCV2 and PCV3 viremia were significantly higher in POS-PPV7 serum samples than in NEG-PPV7 samples (13, 14, 17, 24). In addition, PPV7, PCV2, and PCV3 viral loads were correlated (17, 24). Thus, it was speculated that PPV7 is an important cofactor in PCV2 and PCV3-associated diseases. The current study showed that PRRSV positivity was higher in POS-PPV7 samples than in NEG-PPV7 samples. In addition, the PRRSV copy number was statistically significantly higher in POS-PPV7 samples, suggesting that PPV7 may stimulate the replication of PRRSV, similar to the immunosuppression induced by PCV2, PCV3, and PRRSV during infection (25–27), and promote secondary infection or coinfection with other pathogens. Interestingly, PPV7 positivity was higher in POS-PCV2 samples and POS-PRRSV samples than in NEG-PCV2 samples and NEG-PRRSV samples, but the PPV7 copy number was not significantly different in POS-PCV2/NEG-PCV2 and POS-PRRSV/NEG-PRRSV serum samples, indicating that PCV2 and PRRSV infection do not appear to directly stimulate PPV7 replication. Notably, the correlation between the PPV7 copy number and the PCV2 and PRRSV copy numbers was statistically significant. Thus, there may be an association between PPV7 and PCV2 or PRRSV infections, but the related mechanism requires further study.

The mean PPV7 evolutionary rates of *NS1* and *cap* are 8.01 × 10^−4^ and 2.19 × 10^−3^ per site per year, respectively (28). An extremely high nucleotide substitution rate is an important characteristic of PPV7. This study found no deletions or insertions of nucleotides in the *NS1* gene. However, the PPV7 *cap* gene was more prone to nucleotide deletion or insertion and there were eight cap gene lengths, including 1425, 1422, 1419, 1413, 1410, 1404, 1401, and 1392 nt among 128 *cap* gene sequences. Frequent mutations in the *cap* gene would create antigenic variation, induce novel virulence adaption, and contribute to pathoadaptive diversification. While there are differing views of PPV7 phylogenetic analysis, the closest approximation is from a study by Wang et al. (28), in which 45 PPV7 sequences reported to GenBank (some complete and others partial) were analyzed, and two well-differentiated clades were identified (28). Diana et al. divided PPV7 into two clades based on the insertion of five amino acids at the 180–184aa of the VP2 protein (29). In addition, phylogenetic analysis of the *NS1* gene divided PPV7 into six clades, phylogenetic analysis of the *cap* gene divided PPV7 into four clades, and phylogenetic analysis of the complete genome divided PPV7 into six clades. Complete genome phylogenetic analysis revealed that PPV7e and PPV7f are the most widely spread genotypes in the Fujian and Guangdong provinces, respectively. While the PPV7a, PPV7b, PPV7c, and PPV7f subtypes were found in Fujian, the PPV7a-e subtypes were found in Guangdong. Thus, PPV7 has a high genetic diversity in the Fujian and Guangdong regions.

Recombination is one of the evolutionary mechanisms that is critical for the generation of genomic diversity (15). Notably, recombination is also very important for viral evolution and may modify the virulence and pathogenicity of PPV7. The PPV7 KF4 strain was identified as a recombinant, with the 17KWB09 strain from Korean wild boars identified as a major parental virus and the N133 strain from Korean domestic pigs defined as a minor parental strain (30). The PPV7 HBTZ20180519-152 strain isolated from Chinese domestic pigs in 2018 is a recombinant of the major parental JX15-like virus and the minor parental JX38-like strain, both isolated from Chinese wild boars in 2015 (15). The natural recombination of PPV7 in wild boars and domestic pigs suggests that this recombination phenomenon may facilitate the transmission of PPV7 between these species (15). The PPV7 21FJ09 isolate is a putative recombinant of the parental GD291 strain and the AH-PPV720178-1 isolate, both isolated from domestic pigs. Similar to the PPV7 HBTZ20180519-152 recombinant strain (15), the putative recombination site of the 21FJ09 isolate was also located in *NS1*. However, it remains unknown whether natural recombination of PPV7 is more likely to occur in *NS1*.

At present, PPV7 has been found in Asia, North America, South America, and Europe (11, 12, 31, 32). The higher substitution rate of PPV7 vs. PPV1–4 may lead to the emergence of highly adaptive subtypes that can spread quickly in swine herds (28). However, no studies have successfully isolated the virus so it remains difficult to research its pathogenicity and molecular biology.

## Data availability statement

The datasets presented in this study can be found in online repositories. The names of the repository/repositories and accession number(s) can be found in the article/supplementary material.

## Author contributions

XY and AD: conceptualization, resources, supervision, project administration, and funding acquisition. XZ: methodology, formal analysis, data curation, original draft writing and preparation, and visualization. CZ: collection of the samples. SX and ZL: software. XY, AD, XZ, SX, ZL, YC, YL, XH, and GL: validation. AD and XZ: investigation, data curation, and original draft writing and preparation. AD: manuscript review and editing. All authors contributed to the article and approved the submitted version.

## Funding

This study was supported by the Central Guidance on Local Science and Technology Development Fund of Fujian Province (No. 2021L3028) and the Major Project of Science and Technology Program of Fujian Province, China (No. 2019NZ09005).

## Conflict of interest

The authors declare that the research was conducted in the absence of any commercial or financial relationships that could be construed as a potential conflict of interest.

## Publisher's note

All claims expressed in this article are solely those of the authors and do not necessarily represent those of their affiliated organizations, or those of the publisher, the editors and the reviewers. Any product that may be evaluated in this article, or claim that may be made by its manufacturer, is not guaranteed or endorsed by the publisher.
